# Mycobacterial infections in a large Virginia hospital, 2001-2009

**DOI:** 10.1186/1471-2334-11-113

**Published:** 2011-05-05

**Authors:** Gowri Satyanarayana, Scott K Heysell, Kenneth W Scully, Eric R Houpt

**Affiliations:** 1 Department of Medicine, Charlottesville, VA 22908, USA; 2 Division of Infectious Diseases and International Health, Charlottesville, VA 22908, USA; 3 Department of Public Health Sciences, University of Virginia, Charlottesville, VA 22908, USA

## Abstract

**Background:**

In areas where both tuberculosis (TB) and nontuberculous mycobacteria (NTM) are prevalent, descriptive studies of the clinical features of individual mycobacteria are needed to inform clinical triage.

**Methods:**

We queried the University of Virginia Clinical Data Repository for all mycobacterial infections from 2001-2009.

**Results:**

Of 494 mycobacterial infections in 467 patients there were 22 species. Patients with pulmonary Tb were more likely to be reported as immigrants (p < 0.001) and less likely to have a predisposing risk factor for NTM (pre-existing lung disease or host predisposition; p = 0.002). Review of chest CT scans revealed that TB infection was more likely to exhibit cavities and pleural effusion than NTM infection (p < 0.05). Among NTM infections *M. kansasii*, *M. xenopi*, and *M. fortuitum *were more likely than MAC to have cavities. There were at least 83 patients that met criteria for NTM lung disease and these were caused by 9 species. *M. abscessus *infection was associated with cystic fibrosis and *M. xenopi *infection was associated with male gender.

**Conclusions:**

In our center mycobacterial infections were common and of diverse species. Immigrant status, cavities, and effusion were associated with TB vs. NTM.

## Background

A better understanding of the epidemiology of mycobacterial infections leads the research agenda issued by national NTM guidelines [1]. NTM infections are usually not reported to public health departments, thus data on incidence and species distribution are sparse. However there have been suggestions that NTM disease is increasing in some areas [2,3], making the demography and species distribution of these infections important to understand. The mid-Atlantic and southeastern United States are known to be high incidence regions for mycobacteria, likely related to a high prevalence of NTM in local soils [4]. The University of Virginia hospital is a tertiary referral center that receives patients from across the region. Thirty years have passed since the last systematic report of mycobacterial infections at our institution [5]. Thus we sought to describe the epidemiology, clinical characteristics, and radiology of all mycobacterial infections seen at our hospital from 2001-2009. To our knowledge, this is among the largest radiographic series of pulmonary mycobacterial infections reported, with 244 chest CT scans available for analysis.

The clinical approach to mycobacterial infection is vexing until speciation is confirmed, a process that can take weeks. In particular, when faced with positive acid-fast bacilli smears or positive growth, the dilemma emerges of whether to treat empirically for TB or an NTM or wait. Rapid nucleic acid amplification tests for Tb can be useful in this scenario but are only approved for sputum or bronchoalveolar lavage material and are not always available. The decision to defer treatment for TB risks ongoing infectiousness and clinical deterioration, while presumptive treatment for TB incurs significant polypharmacy and may trigger contact investigations. We therefore sought to identify clinical parameters that could aid in predicting TB infection over other NTM in our setting.

## Methods

### Clinical data collection

The University of Virginia Health System is a 577-bed hospital with approximately 28,000 inpatient admissions and 112,000 outpatient visits a year. We queried the University of Virginia Clinical Data Repository for laboratory results that included the terms "mycobacteria" or "acid fast bacilli" from January 01, 2001 through August 31, 2009. Clinical information sought for each patient included sex, age, other medical conditions, and site of infection. Site of infection was categorized into five possibilities, including lung (including sputum, bronchoalveolar lavage, and pleural fluids); skin, soft tissue, and bone; multiple/disseminated, if two or more specimens came from two or more sites (peripheral blood, stool, sputum, BAL, catheters, synovial fluid); lymphatic; and other sites, which included genitourinary, pericardium, catheters and surgical wounds. Repeat positive cultures with the same species in the same patient from the same site were considered one infection during this study period. Chest CT scans obtained within six weeks of mycobacterial specimen collection from sputum, bronchoalveolar lavage (BAL) or lung tissue were analyzed. CT scan reports were read for description of bronchiectasis, nodules, cavities, or pleural effusion. Scans without any such findings were reported as having none. The study was approved by the University of Virginia Institutional Review Board.

### Definitions

American Thoracic Society (ATS)/Infectious Disease Society of America (IDSA) guidelines were applied to patients with pulmonary mycobacteria (1). We adhered to the ATS/IDSA definition of two or more sputa or one bronchoalveolar lavage specimen that was culture positive for NTM, plus chest CT findings of bronchiectasis, nodules, or cavitary lesions, plus no other alternative explanation for disease after chart review.

### Species identification

Clinical specimens were prepared by standard protocol and inoculated for culture on Lowenstein-Jensen slants and in liquid media throughout the study period. Identification of mycobacterial species was made by gene probes (AccuProbe, Gen-Probe, San Diego, CA) for *M. avium *complex (MAC), *M. gordonae *and *M. tuberculosis *complex. Speciation not determined by these methodologies was resolved by sequencing of the 16S rDNA or ITS gene. NTM drug susceptibilities were obtained as referral tests.

### Statistics

Species distribution by site of infection was described with simple frequencies and proportions. Annual trends in mycobacterial infection were examined by Pearson correlation. Means and medians were compared using t-test and Mann-Whitney U test, respectively. Categorical variables were analyzed with chi-square or Fisher's exact test. Proportion of a species for a site was analyzed against other species by comparing the proportion's 95% confidence intervals, which had to be non-overlapping and p < 0.05 by Fisher's exact test to meet statistical significance. Bivariate logistic regression was used to determine risk factors for TB among all subjects with pulmonary specimens and separately among those with CT scans. All p values were two-tailed. Data were analyzed with SPSS Statistics 17.0 software.

## Results

### Mycobacterial isolates and species distribution

**Figure 1:**
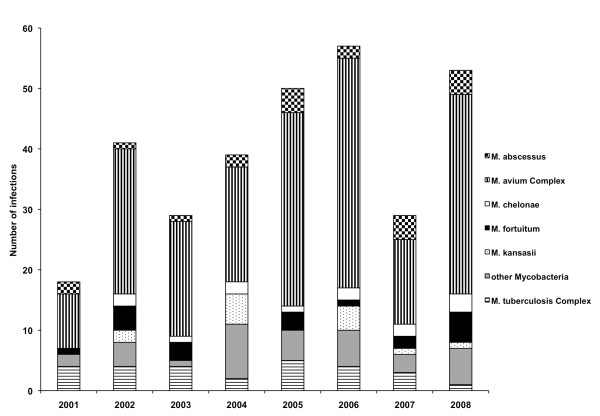
**Mycobacterial infections over time at UVA Hospital**. All mycobacterial infections at University of Virginia Hospital from 2001-2008 were analyzed by year. p = 0.11 for increase over time.

Query of the University of Virginia Clinical Data Repository yielded 2,823 "hits" for mycobacteria or acid-fast bacilli. These results were then cleaned manually and found to represent 494 unique mycobacterial infections from 467 different patients (due to several mixed infections) involving at least 22 species (Table 1). Ninety-eight percent of patients had complete clinical information. Numbers of clinically significant infections were examined over time for each calendar year (Figure 1). *M. avium *complex (MAC) was the most prevalent species each year.

**Table 1:** Site of infection for mycobacterial isolates, 2001-2009 (n = 494)

Species (n; %)	Lung (n)	Skin, soft tissue, bone (n)	Multiple/ Disseminated (n)	Lymphatic (n)	Other site^a ^(n)
TB (37)	22	2	5	3	5
MAC (202)	157	11	18	11	5
*M. gordonae *(142)	137	0	0	0	5^b^
*M. abscessus *(22)	16	3	1	1	1
*M. fortuitum *(19)	12	5	1	0	1
*M. chelonae *(14)	7	3	3	0	1
*M. kansasii *(14)	14	0	0	0	0
*M. marinum *(8)	0	8	0	0	0
Other NTM (36)^c^	29	3	2	0	2
Total (494)	394	35	30	15	20

^a^Other sites included genitourinary, pericardium, catheters, and wounds,

^b^*M. gordonae *from other sites included 2 from urine and 2 from stool.

^c ^Other NTM from lung included 1 *M. arupense*, 1 *M. interjectum*, 2 *M. kubicae*, 1 *M. margeritense*, 1 *M. mucogenicum*, 2 *M. nebraskense*, 1 *M. paraffinicum*, 3 *M. peregrinum*, 2 *M. scrofulaceum*, 2 *M. simiae*, 1 *M. szulgai*, 5 *M. terrae*, 1 *M. triplex*, 1 unidentifiable NTM, 5 *M. xenopi*. Other NTM from skin, soft tissue, and bone contained 1 *M. smegmatis*, 1 *M. peregrinum*, and 1 *M. xenopi *infection. Other NTM from multiple/disseminated sites included 1 *M. immunogenum *and 1 *M. haemophilum*. Other NTM from Other sites included 1 *M. fluoranthenivorans *catheter infection and 1 *M. terrae *culture from urine.

### Frequency and site of mycobacterial infection

We then examined the different anatomic sources of mycobacterial infection. For this analysis we included *M. gordonae *because these infections can confound clinical decision-making and must be considered until speciation is known. Lung was the major site of infection (n = 394, 80% of total), followed by skin, soft tissue, or bone (n = 35, 7%), and multiple sites/disseminated infection (n = 30, 6% of total). *M. marinum *infections exclusively derived from skin, soft tissue, or bone and *M. gordonae *was associated with lung, p < 0.05. The majority (75%) of lung mycobacteria were either MAC or *M. gordonae*. Additionally, fully 7% of lung mycobacteria were "other" NTM, constituted by 15 species including 5 *M. xenopi *and 5 *M. terrae *infections. Of note, 9 of the 22 patients with Tb from a lung source had both bronchoalveolar lavage and sputum obtained, and in 5 of 9 instances both were positive, in 4 of 9 instances only sputum was positive, and in no instance was only bronchoalveolar lavage positive.

### Risk factors for pulmonary Tb

Given the frequency of pulmonary mycobacterial infection, we sought to identify clinical factors that could distinguish TB from NTM. Again, since the clinician is presented with a positive pulmonary AFB smear or culture while speciation is pending, it is logical to include *M. gordonae *at this point. Features of individuals with pulmonary Tb versus NTM are shown in Table 2. Patients with pulmonary Tb were more likely to be reported as immigrants (p < 0.001) and less likely to have a predisposing risk factor (p = 0.002). Specific risk factors we included were pre-existing lung disease (defined as any of asthma, alpha-1-antitrypsin, emphysema/COPD, cystic fibrosis, interstitial lung disease, pulmonary alveolar proteinosis, pulmonary fibrosis, sarcoidosis, silicosis), diabetes, HIV, end-stage renal disease, collagen vascular disease, and history of transplant or cancer.

**Table 2:** Features of patients with pulmonary *M. tuberculosis *and nontuberculous mycobacteria infection.

Characteristic	Total n = 363 (%)	NTM n = 342 (%)	TB n = 21 (%)	*P *value
Age (mean yrs) [SE mean] Age≥60 yrs	57.2 [1.0] 186 (51)	57.6 [0.9] 176 (51)	50.0 [5.5] 10 (48)	p = 0.19 p = 0.73
Gender: Male	181 (49)	171 (50)	10 (48)	p = 0.83
Immigrant	17 (5)	3 (1)	14 (67)	p < 0.001
Predisposing risk factor for NTM**	261 (72)	252 (75)	9 (43)	p = 0.002
CT scan				
Cavities	36 (14)	31 (13)	5 (38)	p = 0.01
Bronchiectasis	58 (24)	56 (24)	2 (15)	p = 0.74
Nodules	111 (45)	103 (45)	8 (61)	p = 0.23
Effusion	15 (6)	11 (5)	8 (61)	p = 0.005

*Note total number of pulmonary patients (n = 363) is lower than total number of lung infections (n = 394) because of mixed infections.

**Predisposing risk factors for NTM = pre-existing lung disease (COPD, Cystic fibrosis, asthma, alpha-1-antitrypsin, interstitial lung disease, pulmonary alveolar proteinosis, pulmonary fibrosis, sarcoidosis, and silicosis) or host predisposition (diabetes, end-stage renal disease, collagen vascular disease, cancer, HIV, transplantation).

### Chest CT findings in pulmonary mycobacterial infections

**Figure 2:**
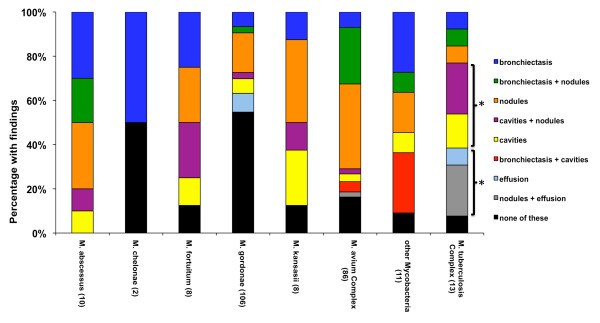
**Chest CT scan findings in patients with pulmonary mycobacterial infection (n = 244)**. CT scans were performed within 6 weeks of pulmonary specimen and radiologist reports read for findings of bronchiectasis, nodules, cavities and/or effusion. (A) All 244 CT scans could be binned into categories of either bronchiectasis, bronchiectasis + nodules (B+N), nodules, cavities + nodules (C+N), cavities, bronchiectasis + cavities (B+C), effusion, nodules + effusion (N+E). *, p < 0.05 for *M. tuberculosis *rate of cavities or effusion vs. all NTM.

**Figure 3:**
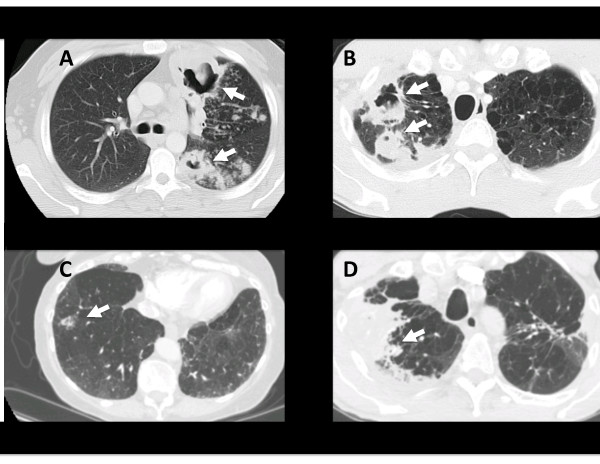
**Representative CT images for mycobacterial infections**. Shown are images for (A) *M. tuberculosis*, with cavities (arrows), in a 35 y.o. foreign-born individual with no predisposing risk factor for NTM, (B) *M. kansasii*, with cavity (arrow), in a 51 y.o. male with COPD, (C) *M. avium*, with nodule (arrow), in a 70 y.o. female with COPD, and (D) *M. xenopi*, with 7 cm peripheral wedge shaped consolidation (arrow) with air cavities, in 66 y.o. male with COPD.

Among the 363 patients with pulmonary mycobacterial infection 244 had chest CT scans performed within 6 weeks of the specimen. The radiologists' findings were read for description of bronchiectasis, cavities, and nodules, since these are the key radiographic metrics of NTM lung disease. The frequency of pleural effusions was also examined. Patients with TB were more likely to have cavities (p = 0.01) and effusions (p = 0.005) than patients with NTM (Figure 2 and Table 2). Within NTM, *M. fortuitum*, *M. kansasii*, and *M. xenopi *were more likely to exhibit a cavitary pattern than MAC infection (3/8, 3/9, and 4/5 vs. 9/97, respectively, p < 0.05). Otherwise there was no statistically discernable pattern for a given species and it was common for chest CT scans to reveal nodules and/or bronchiectasis. Respective CT findings are shown for *M. tuberculosis*, *M. kansasii*, *M. xenopi*, and *M. avium *(Figure 3).

### NTM lung disease

We then sought to discern if there were predictors for NTM lung diseases of particular species. We limited this examination to those patients where we could demonstrate all ATS/IDSA criteria for NTM lung disease had been fulfilled (n = 83, Table 3). *M. gordonae *infections were excluded as likely environmental contaminants. NTM lung disease patients with MAC were of a higher median age than those with *M. abscessus *or *M. kansasii *(p < 0.05), owing largely to a high rate of elderly women with MAC (average age 63 vs. 52 years for males, p = 0.05). Cystic fibrosis was statistically associated with both *M. abscessus *lung disease and mixed NTM infections (CF occurred in 3 of 8 *M. abscessus *infections vs. 3 of 72 non-*M. abscessus *infections, and likewise occurred in 3 of 8 mixed infections vs. 3 of 72 non-mixed infections, and, p < 0.05). *M. fortuitum *lung disease was uncommon (3/83) but 66% were associated with cancer. No other host characteristics were statistically associated with particular mycobacterial species.

**Table 3:** Host characteristics among patients meeting ATS/IDSA criteria for NTM lung disease

	Demographics			Lung disease			Host predisposition			
**Organism (N)**	**Age (median)**	**Sex** **M:F**	**Immigrant n (%)**	**COPD** **n (%)**	**Cystic Fibrosis** **n (%)**	**Other lung dz^a^** **n (%)**	**DM/ESRD/CVD** **n (%)**	**Cancer** **n (%)**	**HIV** **n (%)**	**Transplant** **n (%)**

Total meeting ATS/IDSA criteria (83)	59	28:55	1 (1)	19 (23)	6 (7)	13 (16)	11 (13)	10 (12)	3 (4)	5 (6)

MAC (57)	62	15:42	0	10 (18)	3 (5)	8 (14)	8 (14)	8 (14)	2 (4)	2 (4)
*M. kansasii *(4)	43	3:1	0	2 (50)	0	1 (25)	0	0	0	0
*M. xenopi *(4)	70	4:0^b^	0	2 (50)	0	0	0	0	1 (25)	0
*M. abscessus *(3)	43	0:3	1 (33)	1 (33)	0	1 (33)	0	0	0	1 (33)
*M. fortuitum *(3)	62	0:3	0	0	0	1 (33)	1 (33)	2 (66)	0	0
Mixed^c ^(8)	42	4:4	0	2 (50)	3 (38^d^)	1 (13)	2 (25)	0	0	2 (25)
Other^e ^(4)	59	2:2	0	2 (50)	0	1 (25)	3 (75)	0	0	0

^a^Other lung disease = asthma, alpha-1-antitrypsin, interstitial lung disease, pulmonary alveolar proteinosis, pulmonary fibrosis, sarcoidosis, and silicosis.

^b^*M. xenopi *associated with male gender, p = 0.01.

^c^Mixed included 4 MAC + *M. abscessus *infections, 1 MAC + *M. fortuitum *infection, 1 MAC + *M. terrae *infection, and 1 *M. abscessus *+ *M. chelonae *infection. 5 of 8 *M. abscessus *infections were mixed, vs. 3 of 75 non-abscessus infections, p < 0.05.

^d ^Cystic fibrosis was statistically associated with mixed infections vs. non-mixed infections, p < 0.05.

^e ^Other infections included 1 each of *M. chelonae*, *M. simiae*, *M. szulgai*, and *M. terrae*.

DM = diabetes mellitus. ESRD = end-stage renal disease. CVD = collagen vascular disease.

### Antibiotic susceptibility results

Susceptibilities were performed on 24 (12%) MAC isolates and 23 (96%) were susceptible to clarithromycin [1,6]. Susceptibilities were performed on 10 (45%) *M. abscessus *isolates and were reported as susceptible to clarithromycin in 9 (90%), to amikacin in 9 (90%), and to cefoxitin in 3 (30%).

## Discussion

This current study of mycobacterial infections is drawn from a geographic area prevalent for both NTM and TB and spans nearly a decade. The study describes important epidemiology regarding demography, speciation, and pathogenesis, with particular attention paid to pulmonary mycobacterial infections and chest CT scan findings. Given the lack of mandatory reporting of NTM, studies from large referral institutions such as this are critical to understand regional patterns or emerging trends. We observed a non-statistically significant rise in the number of mycobacterial isolates over the last decade, significant species diversity, with more total cases of pulmonary NTM than during an equivalent time period from our institution thirty years ago [5]. Estimation of the true change in incidence is difficult from these data given alterations in population referral, potentially heightened clinician suspicion of NTM, and improved diagnostics, however our findings complement trends in other parts of the world that suggest increasing rates of NTM disease and species diversity [2,3,7].

Among patients meeting criteria for NTM lung disease, MAC remained the overwhelming pathogenic species, consistent with previous findings at our institution and typical of most NTM epidemiologic reports [8]. After MAC we observed significant diversity of 8 other species, as well as 8 mixed infections, which suggests that clinicians should not readily discount a particular species in this region. We looked for risk factors for particular NTM lung species, including age, gender, chronic obstructive pulmonary disease, cystic fibrosis, other lung diseases, diabetes, end stage renal disease, collagen vascular disease, cancer, HIV and transplantation. The only obvious host characteristic that was linked with a particular NTM lung disease was male gender with *M. xenopi *and cystic fibrosis with *M. abscessus*. The latter association has been appreciated before [9] but not usually to this extent, where fully 38% of our *M. abscessus *infections were associated with CF. This may be a feature of our center which is a tertiary referral center for lung transplantation. Of course we may have missed identifying risk factors due to low power for most species besides MAC, but it still suggests to us that in our region mycobacteria do not present stereotypically, thus pathogenesis is likely not species-specific, and no particular species can be easily considered low virulence (as *M. fortuitum *is sometimes considered [1]). Another finding was the relatively low proportion of individuals that strictly met ATS/IDSA criteria for NTM lung disease: 82 of 342 pulmonary NTM infections or 24%. This contrasts with other studies where more than half of all pulmonary NTM patients met these criteria [10]. Our suspicion is that this does not reflect true differences but that our proportion of patients that met criteria for NTM lung disease was an underestimate because we relied tightly on chest CT scans performed at our institution within 6 weeks of the isolate.

Over two thirds of patients with a pulmonary mycobacterial isolate had a CT scan available for analysis, making this one of the largest radiographic series of NTM lung infections available. NTM lung disease has historically been ascribed two radiographic categories: fibrocavitary disease similar to Tb, especially in males, or nodular bronchiectatic disease, especially in females. We could not discern such a distinction and found significant overlap across the species in terms of cavities, nodules, and bronchiectasis regardless of gender. Previous reports have also failed to find a difference in radiologic appearance of NTM infection based on the mycobacterial species [11-13]. However a few trends were revealed. Notably, *M. fortuitum *and *M. kansasii *had similar patterns, with a larger proportion with cavities than the most common NTM species, MAC. Cavitation has been thought to be rare with *M. fortuitum *and the other rapidly growing mycobacteria [14]. Among the patients with *M. fortuitum *and cavities, all had an immunosuppressive condition or pre-existing lung disease that may have contributed. The high frequency of cavities is well known for *M. kansasii *[15,16], often times with thinner walled cavities that Tb, however this latter feature was not reliably described in our radiology reports. Cavities have been reported in up to 50% CT scans in patients with active MAC infection [17], yet others report more bronchiectasis and nodules [13,18,19]. Our series found the latter, with nearly two-thirds of our 86 patients with pulmonary MAC infection and CT scans exhibiting nodules alone or nodules with bronchiectasis. The strength of our series is that it was larger than any of these previous studies.

We acknowledge that our radiologic analysis has limitations. Radiologists may have received information on the microbiologic diagnosis from the clinicians, and this lack of blinding may have led to bias in reporting findings historically associated with the known diagnosis or present on prior scans that we did not review. Furthermore, it may be that patients with characteristic findings on chest radiograph were not sent for chest CT, as may be evidenced by the 21 patients with TB from pulmonary specimens, of whom only 13 had a chest CT available for analysis. On the other hand these limitations are the norm in clinical practice.

The first scenario clinicians face is a positive acid-fast smear or mycobacterial culture and determining a management course while awaiting results of speciation. In comparing risk factors for TB or NTM among patients with pulmonary mycobacterial specimens, likelihood ratio analysis of immigrant status led to post-test probabilities of TB exceeding 80%. The magnitude of this observation may be secondary to reporting bias since a clinician may have been more likely to document in the chart a patient as an "immigrant" if TB was confirmed. Furthermore, the term "immigrant" was not defined or validated. Therefore although our rates of immigrants among Tb and NTM patients may be imperfect they are likely reasonable. Indeed, our proportion of Tb seen among immigrants, 67%, is almost identical to our state's rate of Tb among foreign-born individuals. On the topic of specimen type, our data would emphasize the importance of sputum over bronchoscopy to evaluate for pulmonary Tb, since sputum was as or more sensitive than bronchoalveolar lavage fluid for Tb growth in all 9 instances where both were sent.

Finally, our series revealed a large number of "other" NTM, including at least 3 infections of *M. peregrinum*, *M. terrae*, and *M. xenopi *as well as more than a dozen others. This adds important detail to the known epidemiology of NTM. For instance, *M. simiae *has been appreciated mostly from the southwestern United States [1], but not our region. Additionally, the NTM guidelines describe *M. terrae *as "potentially pathogenic," which we would strongly support. And *M. xenopi *is reported as rare in the US, although we observed 5 infections with some extensive disease.

## Conclusions

In summary, we found a substantial number and diversity of mycobacteria isolated from patients at our center throughout the past decade. Series such as this from other centers are welcomed to better inform the complex and emerging epidemiology of mycobacterial infections.

## Competing interests

The authors declare that they have no competing interests.

## Authors' contributions

GS participated to data acquisition, analysis, and drafting of the manuscript. KS contributed to data acquisition. SH contributed to data analysis and writing. EH contributed to study design, data analysis, and writing. All authors read and approved the final manuscript

## Pre-publication history

The pre-publication history for this paper can be accessed here:

http://www.biomedcentral.com/1471-2334/11/113/prepub
